# Recent Advances in C-F Bond Cleavage Enabled by Visible Light Photoredox Catalysis

**DOI:** 10.3390/molecules26227051

**Published:** 2021-11-22

**Authors:** Lei Zhou

**Affiliations:** School of Chemistry, Sun Yat-Sen University, Guangzhou 510006, China; zhoul39@mail.sysu.edu.cn

**Keywords:** visible light, radical, C-F bond, cleavage, photoredox

## Abstract

The creation of new bonds via C-F bond cleavage of readily available per- or oligofluorinated compounds has received growing interest. Using such a strategy, a myriad of valuable partially fluorinated products can be prepared, which otherwise are difficult to make by the conventional C-F bond formation methods. Visible light photoredox catalysis has been proven as an important and powerful tool for defluorinative reactions due to its mild, easy to handle, and environmentally benign characteristics. Compared to the classical C-F activation that proceeds via two-electron processes, radicals are the key intermediates using visible light photoredox catalysis, providing new modes for the cleavage of C-F bonds. In this review, a summary of the visible light-promoted C-F bond cleavage since 2018 was presented. The contents were classified by the fluorosubstrates, including polyfluorinated arenes, *gem*-difluoroalkenes, trifluoromethyl arenes, and trifluoromethyl alkenes. An emphasis is placed on the discussion of the mechanisms and limitations of these reactions. Finally, my personal perspective on the future development of this rapidly emerging field was provided.

## 1. Introduction

The C-F bond is the strongest covalent single bond that carbon can form. The strength of the C-F is among the reasons why organofluorine compounds have found wide applications in diverse fields [1,2,3,4]. The global production of fluorochemicals during 2018 was 4.2 million tons with a marker value of 24.6 billion USD [5]. Man-made fluorinated organics usually contain many fluorine substituents and may even be fully fluorinated [6]. Selective defluorinative functionalization of readily available per- or oligofluorinated reagents provides an appealing approach to access partially fluorinated synthetic intermediates, especially those are not easily made by the conventional C-F bond formation protocols. Consequently, the development of new synthetic methods for C-F bond cleavage has attracted considerable attention in the past decades [7,8,9,10,11,12,13,14]. In light of the environmental concerns, fluorinated organics are difficult to be degraded and tend to accumulate [15]; the conversion of these persistent organic pollutants into high-valued organofluorine compounds is also of great significance.

Two main synthetic strategies have been developed for the C-F bond cleavage: one is the direct C-F bond functionalization, and the other is based on β-F elimination. The direct C-F bond functionalization can be achieved by (1) forming a carbon-metal bond via the addition of the C-F bond to an electron-rich metal center [10]; (2) generating carbocation via main-group Lewis acids-mediated fluoride abstraction [8]; and (3) two-electron reduction using low-valent metals or electrochemical methods to give carbon ions [9,16]. While the β-F elimination approach depends on the formation of either a carbon ion or a carbon-metal bond adjacent to the C-F bond [14]. Although organometallic species, carbon cations, and carbon ions have been extensively studied as the key intermediates for the C-F bond cleavage, radicals, which are among the common intermediates in organic synthesis, have rarely involved in C-F bond cleavage reactions.

**Scheme 1:**
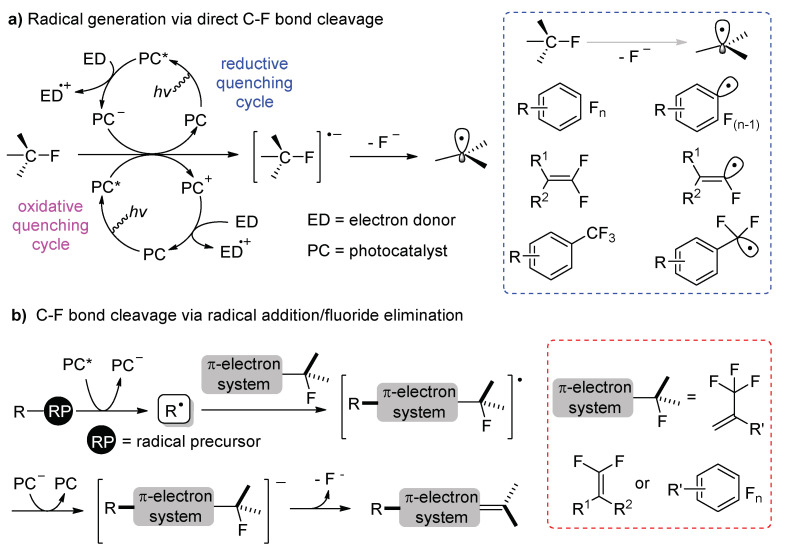
Two general activation modes for C-F bond cleavage by visible light photoredox catalysis.

**Scheme 2:**
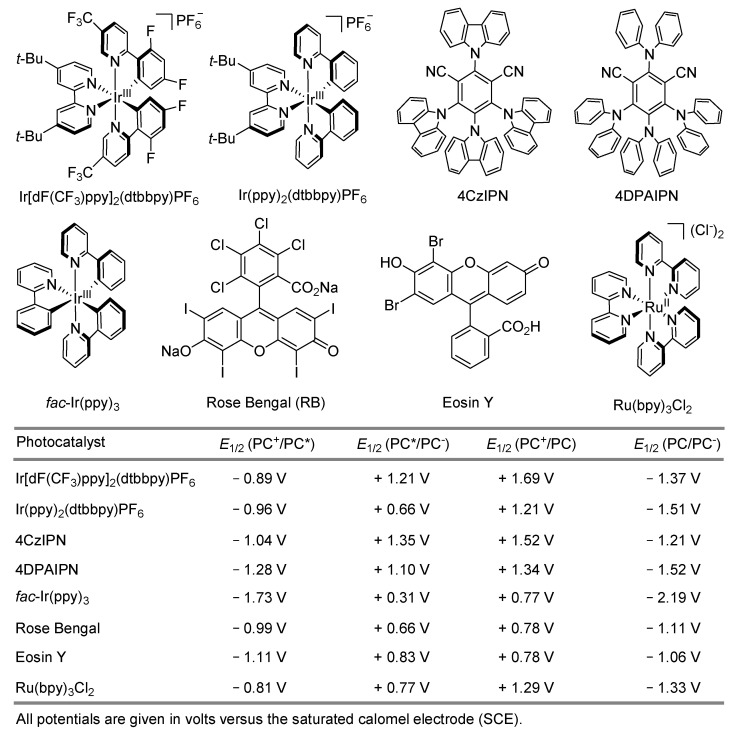
Typical photocatalysts for C-F bond cleavage and their redox potentials.

Visible light photoredox catalysis has emerged as a versatile platform for organic synthesis under exceptionally mild conditions over the past decade [17,18,19,20]. Due to the unique redox properties of photocatalysts, the direct C-F bond cleavage enables the generation of carbon radicals by either reductive quenching or oxidative quenching of the excited photocatalyst (Scheme 1a). In the reductive quenching cycle, the excited photocatalyst was first single-electron reduced by the electron donor, delivering a stronger reductant PC^−^. Single-electron transfer (SET) between PC^−^ and fluorosubstrate generates highly reactive radical anion, which eliminates fluoride to give a carbon radical. In the oxidative quenching cycle, the excited photocatalyst PC* is proposed to donate an electron to the fluorosubstrates. Since the reducing ability of PC* is lower than PC^−^, the oxidative quenching cycle is less common in photocatalytic C-F bond cleavage reactions. Generally, the presence of electron-withdrawing groups and π-systems in the fluorosubstrates are favorable for accepting an electron from the photocatalyst. This activation mode is suitable for the C-F bond cleavage of polyfluorinated arenes, *gem*-difluoroalkenes, and trifluoromethyl arenes, producing oligofluorinated aryl radicals, monofluoroalkenyl radicals, and α,α-difluororobenzylic radicals, respectively. Another key feature of photoredox catalysis is its potential to crossover from a radical to a polar pathway during the overall catalytic process [21], which can be applied to the C-F bond cleavage through β-fluoride elimination. As shown in Scheme 1b, the single-electron oxidation of radical precursors by the excited photocatalyst would give radical R^•^ and PC^−^. The addition of R^•^ to the π-electron system of fluorosubstrate gives carbon radical, which can be single-electron reduced by PC^−^ to form carbon ions to facilitate the fluoride elimination. Trifluoromethyl alkenes are typical substrates for such transformation. Recently, the C-F bond cleavage of *gem*-difluoroalkenes and highly fluorinated arenes has also been reported via this addition-elimination process, depending on photocatalysts and radical precursors that used. Typical photocatalysts for C-F bond cleavage and their redox potentials are shown in Scheme 2 [22].

Since the first photocatalytic hydrodefluorination (HDF) of perfluoroarenes was reported by Weaver in 2014 [23], the use of visible light photoredox catalysis to promote the inert C-F bond cleavage continues to attract the most attention. We have previously published a book chapter, namely visible light-mediated C-F bond activation, which summarized the advances in the area from 2014 to 2017 [24]. Now, the level of interest in this topic is greater, and the expansion into the synthesis of various organofluorine compounds during the past 3–4 years has been achieved. Therefore, we feel justified in again reviewing the topic with the same focus that characterized our earlier review. Although there have been several reviews on C-F bond cleavage recently, each review has its specific focus, such as C-F bond activation by phosphorus compounds [25], or by metalloenzymes [26], C-F functionalization of trifluoromethyl groups [27,28], catalytic enantioselective functionalization of allylic C-F bond [29], transition metal-catalyzed C(sp^2^)-F cleavage [30], and deconstructive modes of halodifluoromethyl reagents [31]. This review focuses on the C-F bond cleavage enabled by visible light photoredox catalysis since 2018, classified by the types of fluorosubstrates [32,33].

## 2. Polyfluorinated Arenes

**Scheme 3:**
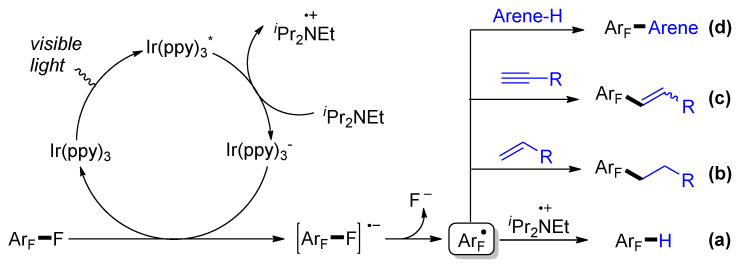
Photocatalytic C-F bond functionalization of polyfluorinated arenes: (**a**) hydrodefluorination; (**b**) alkylation; (**c**) alkenylation; (**d**) arylation.

Simple polyfluorinated arenes are inexpensive feedstocks produced by the Halex process [34]. The Weaver group has reported the replacement of unwanted fluorine atom of perfluorinated (het)arenes by hydrogen (Scheme 3a) [23], alkyl (Scheme 3b) [35], alkenyl (Scheme 3c) [36], and electron-rich aryl groups (Scheme 3d) [37] through photocatalytic C-F bond cleavage. Upon visible light irradiation, the excited *fac*-Ir(ppy)_3_^*^ is single-electron reduced by *^i^*Pr_2_NEt (DIPEA), providing the stronger reductant *fac*-Ir(ppy)_3_^−^. The reduction of perfluorinated arenes by *fac*-Ir(ppy)_3_^−^ produces perfluoroaryl radical anion, followed by the extrusion of fluoride to form perfluoroaryl radical. The resulting radical Ar_F_^•^ can either abstract H-atom from the amine radical cation to give the hydrodefluorination (HDF) product, or undergo addition to π systems, including alkenes, alkynes, and arenes. To suppress the competitive hydrogen abstraction, excess amounts of radical acceptors were usually required.

**Scheme 4:**
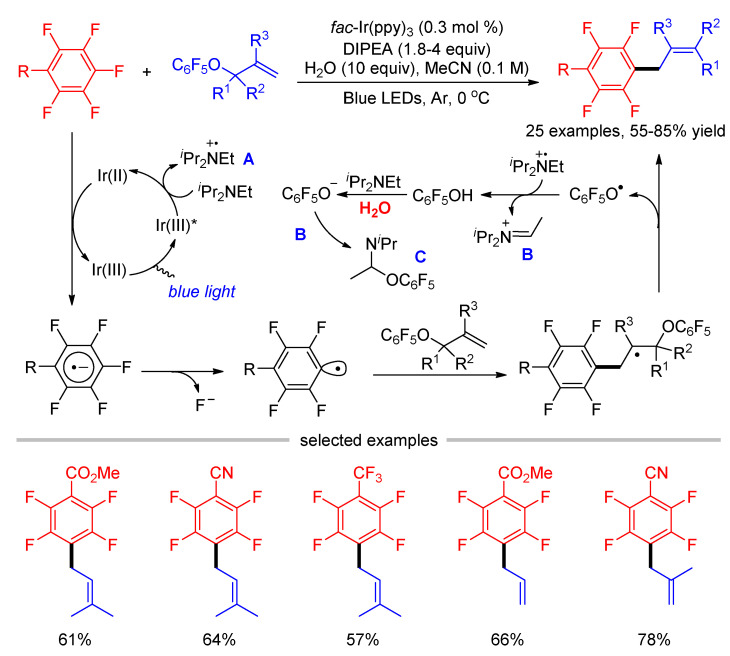
Photocatalytic defluoro–allylation of perfluoroarenes.

As an extension of these works, Weaver described a photocatalytic allylation of electron-deficient perfluoroarenes using perfluorophenyl allyl esters as the radical acceptors in 2018 (Scheme 4) [38]. The use of perfluorophenyl as the masked group of allyl alcohol is crucial for the reaction, which not only acts as an activating group to promote the radical fragmentation of C-O bond, but also suppressed the competitive [3,3]-sigmatropic rearrangement of allylation reagents. The direct hydrodefluorination was observed as the unavoidable side reaction even using six equivalents of allylation reagents. Interestingly, the authors found that the presence of H_2_O (10 equiv) can significantly improve the product/HDF ratio and accelerate the reaction from 14 to 4 h. Water might play two roles in the reaction, one is the hydration of fluoride to increase the exothermicity of the reaction, and the other is the hydrolysis of the pentafluorophenol. The control experiment indicated C_6_F_5_OH had an inhibitory and deleterious effect on the reaction progress, which was generated in situ during the course of reaction via the hydrogen atom abstraction of phenoxy radical from amine radical cation **A**. The addition of water promoted the formation of phenolate. Thus, C_6_F_5_OH was vanished by forming *N*-acetal species **C** through the addition of phenolate to an iminium ion **B**.

**Scheme 5:**
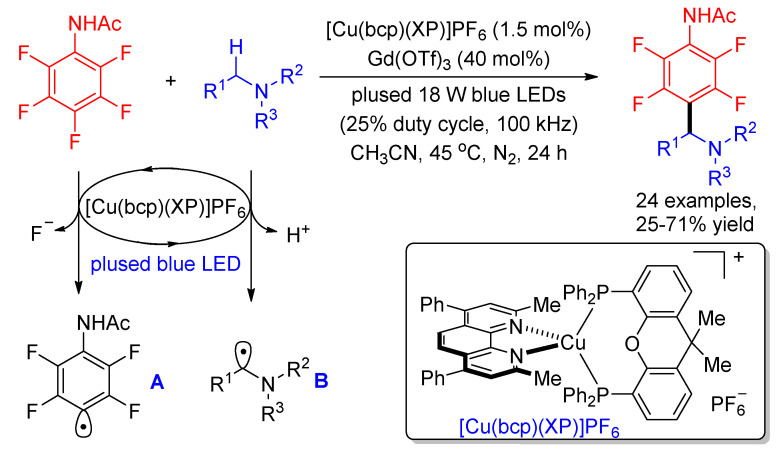
Copper-photocatalyzed dual α-amino C–H/C–F functionalization upon pulsed LED irradiation.

Except for H-atom abstraction and addition to π systems, radical-radical cross coupling is also a possible pathway to trap the perfluoroaryl radicals. Using Ir(ppy)_2_(dtbpy)PF_6_ as the photocatalyst, Xie and Hashmi disclosed the photocatalyzed C(sp^3^)–H multifluoroarylation of tertiary amines with perfluoroarenes via the cross coupling of α-amino alkyl radicals and perfluoroaryl radicals in 2017 [39]. Subsequently, Bissember developed an inexpensive copper-based photocatalyst to replace Ir complex for this reaction (Scheme 5) [40]. Electrochemical characterization of C_6_H_5_NHAc (*E*_1/2_ = −1.60 V vs. SCE) and [Cu(bcp)(XP)]PF_6_ ($E_{1/2}^{I/0}$ = −1.64 V vs. SCE) suggests the generation of both perfluoroaryl radicals **A** and α-amino alkyl radicals **B**. The addition of Lewis acid Gd(OTf)_3_ may lower the redox potentials of C_6_H_5_NHAc and/or tertiary amines by coordination. However, the yields only diminished slightly in the absence of Gd(OTf)_3_. The authors found that an 18 W pulsed blue LED (100 kHz, 25% duty cycle) was an ideal light source, which provided higher yields than the reactions irradiated by a conventional 24 W blue LED.

**Scheme 6:**
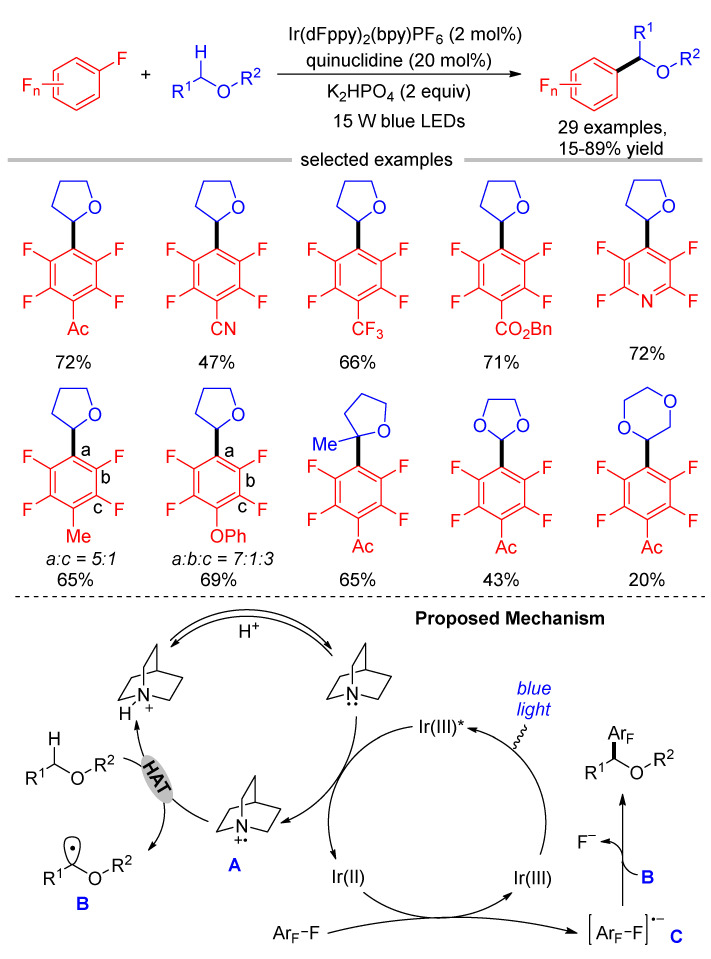
Direct C–H multifluoroarylation of ethers via hydrogen atom transfer.

Although the direct SET oxidation of tertiary amines followed by deprotonation is effective to produce α-amino alkyl radicals, this method is not suitable for the generation of α-alkoxyl alkyl radicals from ethers due to their high oxidation potentials. Using quinuclidine as the H–atom transfer (HAT) catalyst, Xia and Yang reported a visible light-induced multifluoroarylation of ethers with perfluoroarenes (Scheme 6) [41]. The reaction started with the SET oxidation of quinuclidine by the photoexcited Ir(III)*, producing Ir(II) and the radical cation **A**. α-Alkoxyl alkyl radical **B** was generated by the HAT between radical cation **A** and ether, while perfluoroaryl radical anion was formed from perfluoroarene by accepting an electron from Ir(II). Finally, radical–radical cross-coupling of α-alkoxyl radical **B** and perfluoroaryl radical anion **C** with the extrusion of a fluoride ion afforded the products. For polyfluoroarenes bearing a strong electron-withdrawing group, the C-F bond cleavage occurred selectively at the *para* position. However, the regioselectivities were poor when polyfluoroarenes bearing an electron-rich group were used as the substrates. For example, *para-*, *meta-*, and *ortho-* C-F bond functionalization of PhO-substituted polyfluoroarene were observed in the ratio of 7:1:3.

**Scheme 7:**
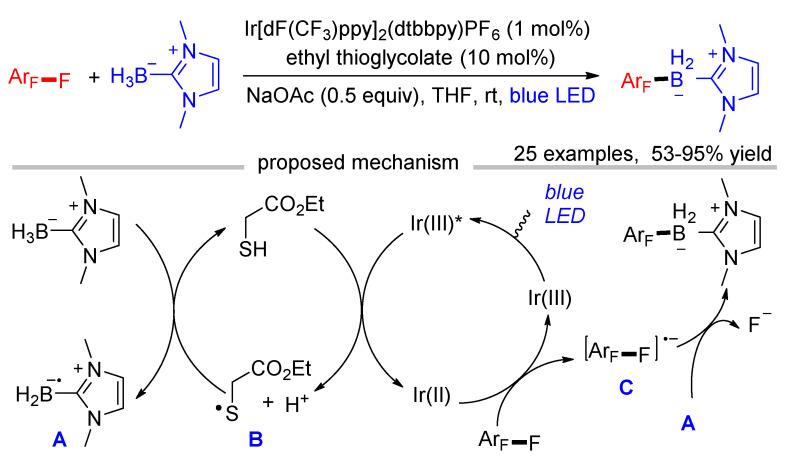
Visible-light-induced defluoroborylation of polyfluoroarenes.

Wu disclosed the defluoroborylation of polyfluoroarenes via the radical–radical cross-coupling pathway with NHC-BH_3_ as the boryl source (Scheme 7) [42]. The bond dissociation energy (BDE) of the B-H bond in NHC-BH_3_ is 72.8 kcal/mol, which is lower than the BDE of the S-H bond (76.1 kcal/mol). Therefore, NHC-boryl radical **A** can be generated through a HAT process mediated by thiyl radical **B**. Single electron oxidation of thiols by the excited photocatalyst followed by the deprotonation in the presence of bases is an efficient method to generate thiyl radicals, while the reduced Ir(II) can activate polyfluoroarenes to give polyfluoroaryl radical anions **C**. This photocatalytic defluoroborylation provides a mild and operationally simple protocol to fluorinated organoboranes, which are versatile synthetic precursors for the preparation of valuable fluorinated organic compounds.

**Scheme 8:**
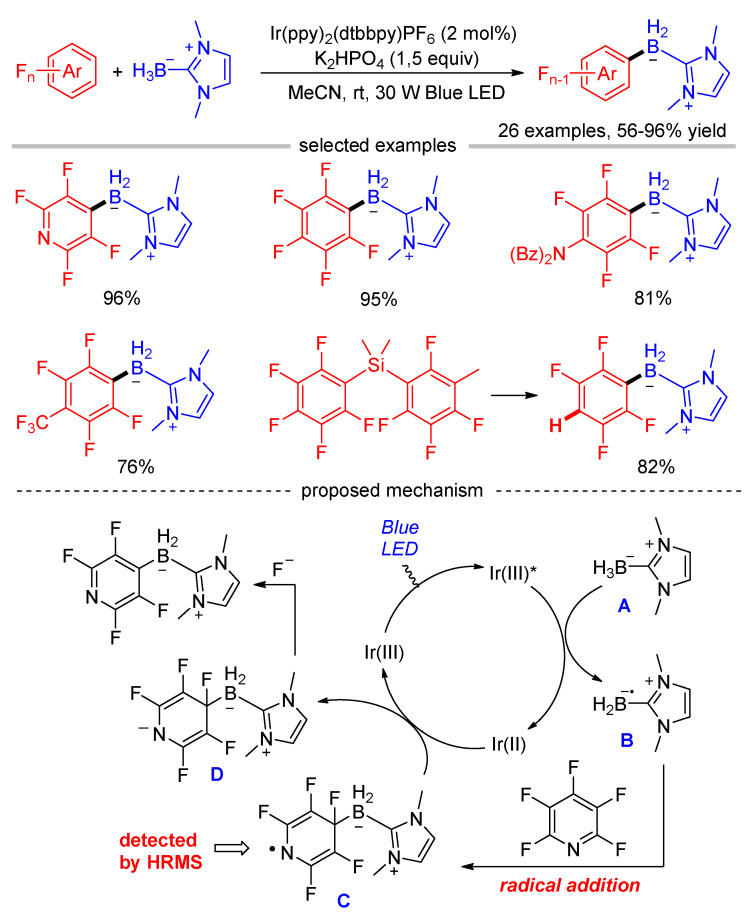
Photocatalytic C–F bond borylation of polyfluoroarenes.

Soon after Wu’s report, Yang and co-workers reported the same photocatalytic C–F bond borylation of polyfluoroarenes without a HAT catalyst (Scheme 8) [43]. Various fluoroarenes, such as perfluoropyridine, perfluoroarene, and 1,2,3,4,5-pentafluorobenzenes bearing an electron-releasing N(Bz)_2_ and electron-withdrawing CF_3_ group, were compatible with the present conditions with good reaction efficiencies. Interestingly, bis(perfluorophenyl)dimethylsilane underwent a desilylation process during the borylation. In this work, NHC-boryl radical was proposed to be generated via the direct SET between photoexcited Ir(III)* and NHC-BH_3_. Because the redox potential of perfluoropyridine ($E_{1/2}^{\mathrm{red}}$ = −2.12 V vs. SCE) is more negative than that of the Ir(II) species (*E*^(IrII/III)^ = −1.51 V vs. SCE), the authors speculated that the reduction of perfluoropyridine to its radical ion by Ir(II) is not favorable. However, the addition of boryl radical to perfluoropyridine would afford the radical intermediate **C**, whose calculated redox potential is −0.24 V, making it an ideal oxidant to accept one electron from Ir(II). Therefore, a mechanism involving radical addition and fluoride elimination was proposed, which was further supported by the detection of radical intermediate **C** by HRMS and the DFT calculations.

**Scheme 9:**
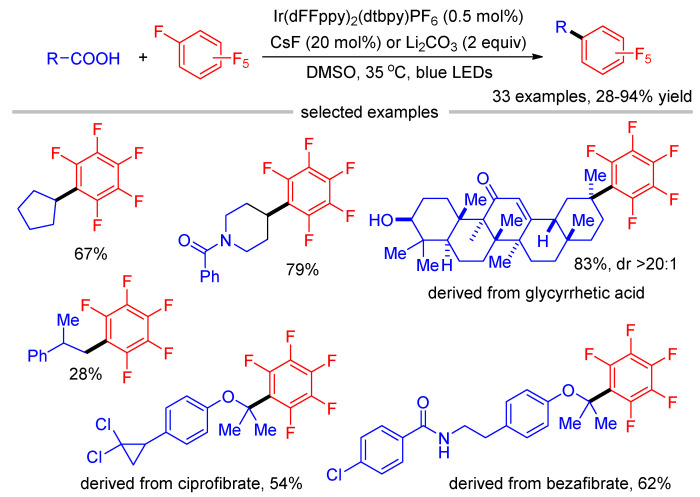
Decarboxylative polyfluoroarylation of alkylcarboxylic acids.

Recently, Ritter reported the decarboxylative polyfluoroarylation of alkylcarboxylic acids (Scheme 9) [44]. The authors also tend to support the pathway via radical addition and fluoride elimination rather than radical–radical cross-coupling based on the following facts: (1) the single-electron reduction of perfluoroarene by the iridium photocatalyst is difficult to take place in consideration of their redox potentials (*E*^(IrII/III)^ = −1.32 V vs. SCE and *E*_red_(C_6_F_6_) = −2.85 V vs. SCE); (2) when *^t^*Bu-acetylene, norbornene, and 1,3,5-trimethoxybenzene were used as scavengers, no perfluorophenyl radical adduct was observed; and (3) exclusive fluorine substitution was observed using chloro-fluoro arenes. As a result, the formation of polyfluoroaryl radical anion is less likely. The reaction tolerates a wide range of aliphatic carboxylic acids, as exemplified by the late-stage functionalization of substrates derived from the nature product glycyrrhetic acid as well as drug molecules bezafibrate and ciprofibrate. Because the nucleophilicity of primary radicals is lower than secondary and tertiary carbon radicals, carboxylic acids that result in the formation of primary radicals are poor substrates in this reaction, which is consistent with the radical addition mechanism.

**Scheme 10:**
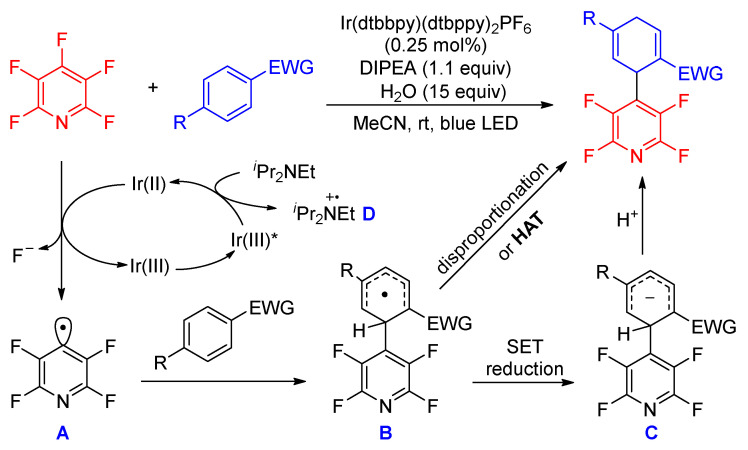
Decarboxylative polyfluoroarylation of alkylcarboxylic acids.

As shown in Scheme 3d, the photocatalytic cross-coupling of perfluoroarenes and electron-rich arenes has been developed for the synthesis of multifluorinated biaryls. The addition of electron-deficient perfluoroaryl radicals to arenes bearing electron-withdrawing groups has been considered to be less efficient. Interestingly, Weaver observed the formation of Brich-type 1,4-cyclohexadiene products in the photocatalytic reactions of perfluoroarenes and electron-deficient arenes (Scheme 10) [45]. However, the direct hydrodefluorination and arylation of perfluoroarenes were still found as the unavoidable background reactions. The addition of 15 equivalent of H_2_O can reduce the number of side products and avoid the darkening of the reaction mixture. Replacing H_2_O by D_2_O led to the partial deuteration products, which suggested there were other potential proton sources in the reaction. Therefore, as with previous photocatalytic arylation of perfluoroarenes, the authors proposed a mechanism involving the formation of doubly allylic radical **B** via the addition of fluoroaryl radical **A** to arenes. SET reduction of **B** and protonation might be the major pathway to form 1,4-cyclohexadienes. Alternatively, the direct HAT of **B** from amine radical cation **D** or disproportionation of **B** is also the possible route.

## 3. *gem*-Difluoroalkenes

**Scheme 11:**
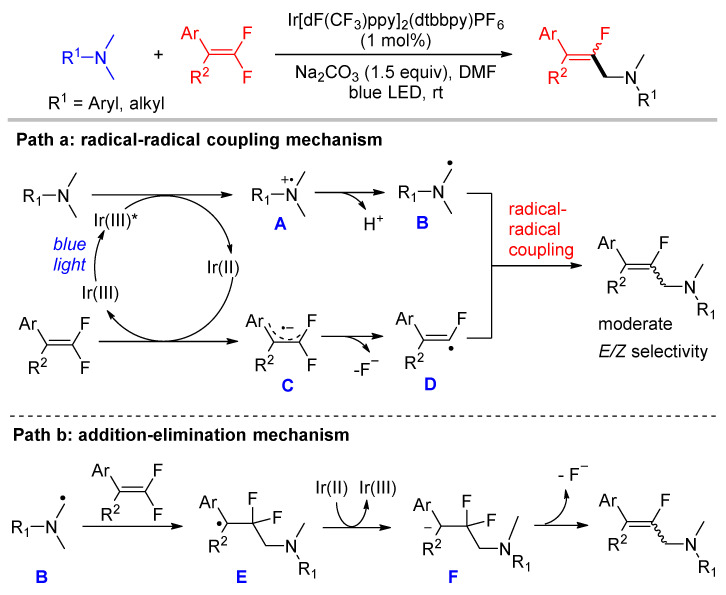
Monofluoroalkenylation of dimethylamino compounds.

Similar to polyfluorinated arenes, the activation of the sp^2^-hybridized C-F bond of *gem*-difluoroalkenes by visible light photocatalysis might occur via radical–radical cross-coupling or the addition-elimination pathway. The first visible light-induced defluorinative monofluoroalkenylation of tertiary amines with *gem*-difluoroalkenes was developed by the Hashmi group in 2016 [46]. The reaction proceeds via a radical–radical coupling mechanism (Scheme 11, path a). Initially, the reductive quenching of excited Ir(III)* by tertiary amine produces radical cation **A** followed by deprotonation to give α-amino alkyl radical **B**, while Ir(III) was reduced to Ir(II). Compared to polyfluorinated arenes, *gem*-difluoroalkenes (for example, diphenyl *gem*-difluoroethylene, $E_{\mathrm{red}}^{1/2}$ = −1.04 V vs. SCE) are easier to be SET reduced by Ir(II), which generate radical anion **C**. The C–F bond fragmentation of **C** affords monofluoroalkenyl radical **D** by the elimination of a fluoride. The DFT calculations indicate that α-amino alkyl radical **B** has a higher SOMO energy than monofluoroalkenyl radical **D**. Therefore, the selective cross-recombination of less reactive α-amino alkyl radical **B** and more reactive monofluoroalkenyl radical **D** could afford monofluoroalkene products according to the “persistent-radical effect”. The radical addition-fluoride elimination pathway (Scheme 11, path b) is less likely because the β-fluoride elimination of several mimic *gem*-difluorophenylethane derivatives bearing acidic benzylic C-H was not observed. The reactions of tertiary amines with unsymmetrical *gem*-difluoroalkenes provided monofluoroalkene products in moderate *E*/*Z* selectivities. The observed stereoselectivity is also consistent with the radical–radical coupling pathway. Subsequently, this strategy was applied to the photocatalytic decorboxylative monofluoroalkenylation of *N*-protected α-amino acids by Fu [47].

**Scheme 12:**
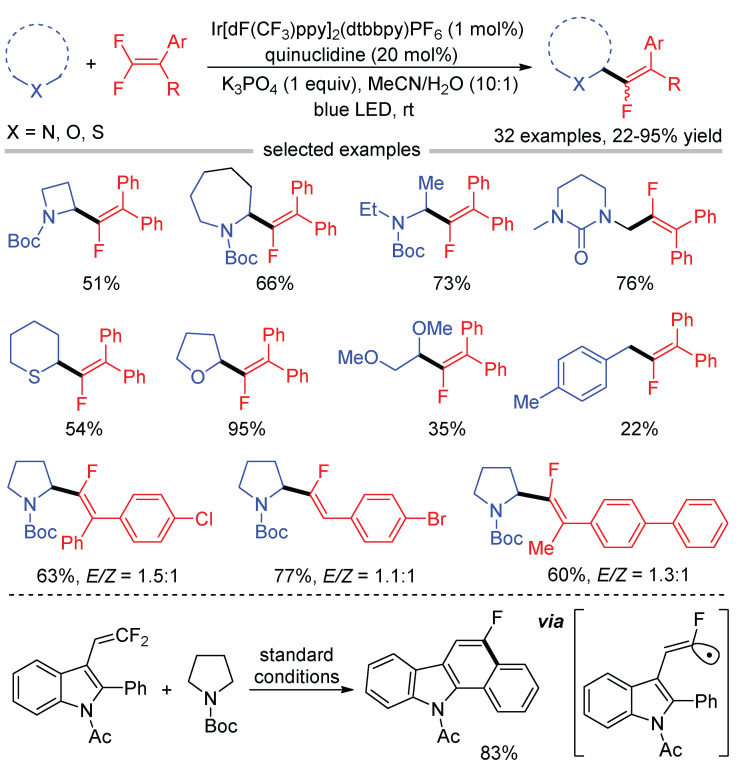
Monofluoroalkenylation of inert C(sp^3^)–H bonds via HAT.

Upon the combination of photocatalysis and HAT catalysis, Wang reported the C(sp^3^)–H monofluoroalkenylation of molecules with high oxidation potentials, such as *N*-Boc protected amines, ethers, thioethers, and xylene (Scheme 12) [48]. The generation of fluoroalkenyl radicals was confirmed by the intramolecular cyclization of *gem*-difluoroalkene bearing 2-phenyl indole motif under standard conditions, which provides strong evidence for the radical cross-coupling mechanism.

**Scheme 13:**
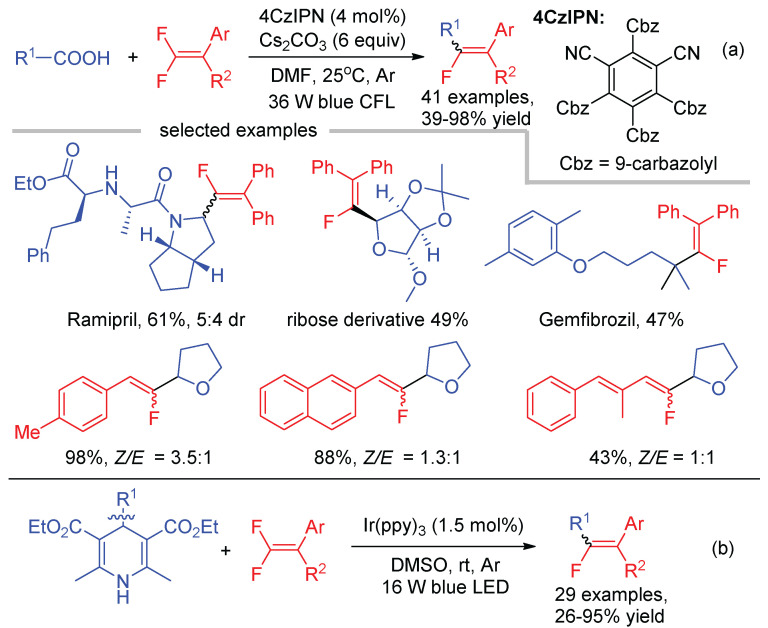
Defluorinative alkylation of *gem*-difluoroalkenes: (**a**) alkyl carboxylic acids as the radical precursors; (**b**) 4-alkyl-1,4-dihydropyridines as the radical precursors.

Alkyl carboxylic acids were less effective radical precursors in Hashmi’s defluorinative coupling with *gem*-difluoroalkenes [46]. Using 4CzIPN as the photocatalyst and a 36 W compact fluorescent lamp (CFL) as the light source, Li and An realized the decarboxylative cross-coupling of *gem*-difluoroalkenes with unactivated carboxylic acids (Scheme 13a) [49]. The reaction tolerates a range of functionalities commonly found in biologically active molecules, which enables the late-stage modification of natural products and medicines, such as the angiotensin-converting enzyme inhibitor Ramipril, ribose derivative, and the fibrate Gemfibrozil. If unsymmetrical *gem*-difluoroalkenes were used as the starting materials, the *E*/*Z* selectivity varied from 1:1 to >20:1 depending on the substituent attached on the C=C bond. The defluorinative alkylation of *gem*-difluoroalkenes was also reported by Zhou and Sun with 4-alkyl-1,4-dihydropyridines as the alkyl radical precursors (Scheme 13b) [50]. Both alkylation reactions were proposed to proceed via the cross-coupling of alkyl radicals and monofluoroalkenyl radicals.

**Scheme 14:**
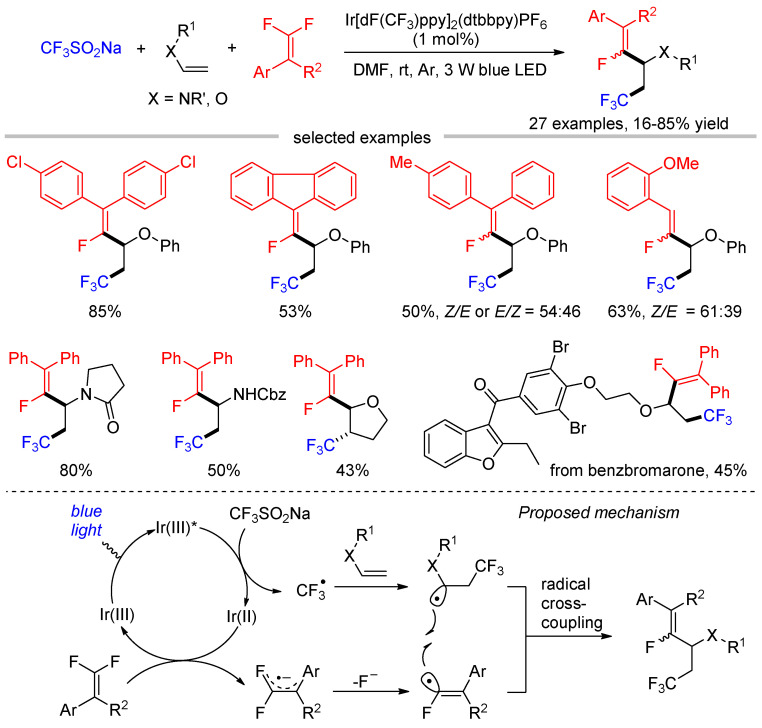
Trifluoromethylation and monofluoroalkenylation of alkenes.

The cross-coupling of monofluoroalkenyl radicals and alkyl radicals can be interrupted by adding another reaction component. Wang disclosed the visible-light-induced trifluoromethylation and monofluoroalkenylation of alkenes through three-component reaction of *gem*-difluoroalkenes, alkenes, and CF_3_SO_2_Na (Scheme 14) [51]. In this reaction, CF_3_ radical, generated by SET oxidation of CF_3_SO_2_Na, would undergo addition to alkenes first. Then, the recombination of the resultant trifluoromethylated alkyl radicals with monofluoroalkenyl radicals delivered the difunctionalization products. The alkenes were limited to enamines and vinyl ethers while non-heteroatom substituted alkenes were not suitable for this transformation.

**Scheme 15:**
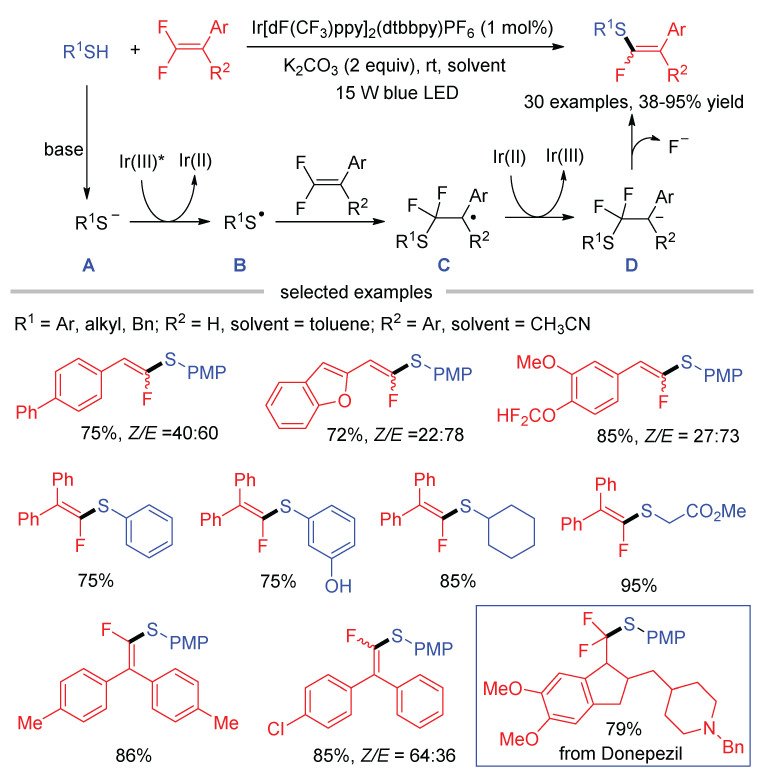
Defluorinative cross-coupling of *gem*-difluoroalkenes with thiols.

The defluorinative cross-coupling of thiols with *gem*-difluoroalkenes for the synthesis of α-fluoro-β-arylalkenyl sulfides was developed by the Xia group (Scheme 15) [52]. The reaction used Ir[dF(CF_3_)ppy]_2_(dtbbpy)PF_6_ as the photocatalyst and K_2_CO_3_ as the base. Despite the base-mediated S_N_V reaction of thiols and *gem*-difluoroalkenes through nucleophilic addition-elimination being a possible pathway [53], the control experiments indicated both photocatalyst and light were necessary for the reaction. In addition, the reaction was completely suppressed in the presence of radical inhibitors. Therefore, a mechanism involving the addition of thiyl radical to *gem*-difluoroalkenes, SET reduction, and β-fluoride elimination was proposed. Non-defluorinative product was obtained when an alkyl–aryl-substituted *gem*-difluoroalkene deriving from donepezil was used as the substrate. This product might be formed by the protonation of intermediate **D** or the direct hydrogen abstraction of radical C from thiols. Consequently, the cross-coupling of thiyl radicals and monofluorovinyl radicals was not considered as an alternative mechanism. Actually, the rate constants for the addition of thiyl radicals onto terminal olefins (*K*_add_) have been measured to be 10^9^ M^−1^ s^−1^ [54], indicating the addition of thiyl radicals to *gem*-difluoroalkenes is a very fast process.

**Scheme 16:**
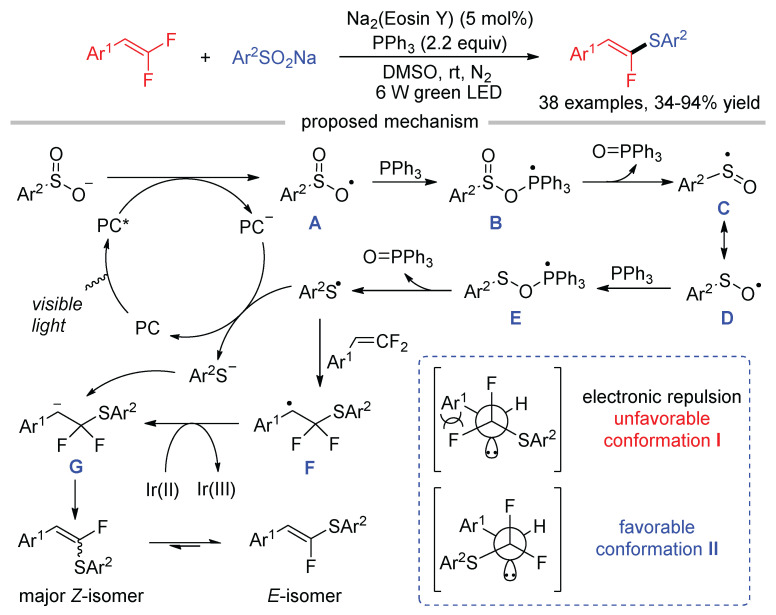
Synthesis of α-fluoro-β-arylalkenyl sulfides by the photocatalytic reaction of *gem*-difluoroalkenes and sodium sulfinates.

Shi reported the generation of thiyl radicals by the deoxygenation of S–O bonds of sodium sulfinates with PPh_3_ as the reductant, which was applied to the synthesis of α-fluoro-β-arylalkenyl sulfides by the reactions with *gem*-difluoroalkenes using organic dye Na_2_(Eosin Y) as the photosensitizer (Scheme 16) [55]. Initially, sodium benzenesulfinate was SET oxidized by excited [Na_2_(Eosin Y)]* to produce an oxygen-centered sulfinic radical **A**. The reaction of **A** with PPh_3_ afforded a phosphoranyl radical **B**, which underwent β-scission to form sulfoxide radical **C** and phosphine oxide. Radical **C** was converted to thiyl radical via a second deoxygenation mediated by PPh_3_. The SET reduction of thiyl radical by reduced [Na_2_(Eosin Y)]^−^ gave thiyl anion and regenerated the ground state [Na_2_(Eosin Y)]. The key carbon ion intermediate **G** for β-fluoride elimination was formed via the direct addition of ArS^−^ to *gem*-difluoroalkenes or the addition of ArS^•^ to *gem*-difluoroalkenes followed by SET reduction. To minimize the electronic repulsion between the fluorine atom and aryl group, *Z*-isomer was obtained as the major product via favorable conformation **II**. Interestingly, the *E*/*Z* selectivity of the products can be improved via *Z* → *E* isomerization under green light irradiation in the absence of a photocatalyst.

## 4. Trifluoromethyl Arenes

**Scheme 17:**
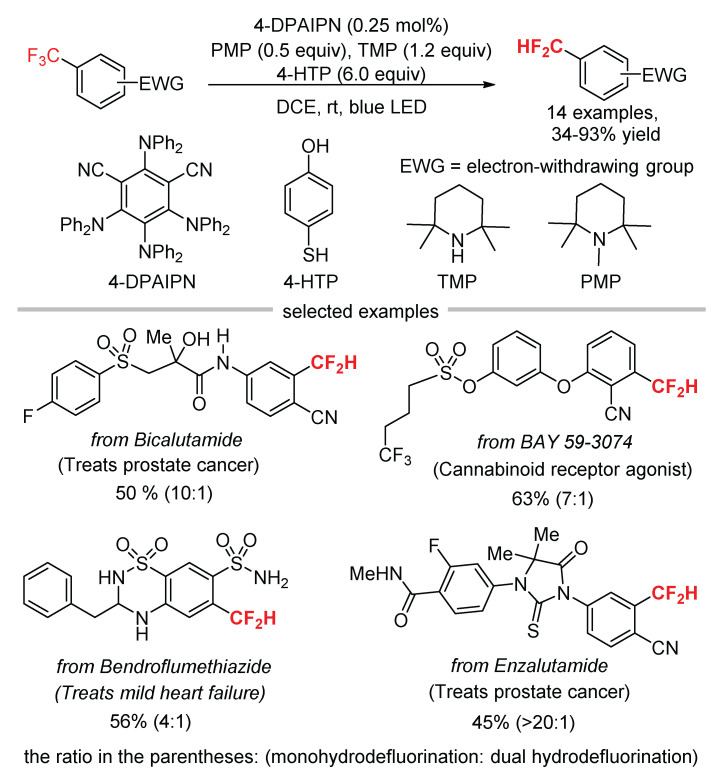
Hydrodefluorination of Trifluoromethylarenes.

Defluorinative functionalization of readily available ArCF_3_ is an ideal protocol to prepare medicinally interesting difluoroalkylated arenes (ArCF_2_–FG). König [56] and Jui [57] independently reported the generation of α,α-difluorobenzylic radicals through single-electron reduction of ArCF_3_ and their subsequent reactions with alkenes. Inspired by these seminal works, Gouverneur developed the hydrodefluorination of trifluoromethylarenes bearing electron-withdrawing groups (EWGs) using 4-DPAIPN as the photocatalyst and 4-hydroxythiophenol (4-HTP) as the hydrogen atom donor (Scheme 17) [58]. Stern–Volmer luminescence quenching experiments indicated the excited photocatalyst was quenched by the combination of 4-HTP and 2,2,6,6-tetramethylpiperidine (TMP). Using a mixture of TMP and 1,2,2,6,6-pentamethylpiperidine (PMP) gave higher yields than the reactions in the presence of either TMP or PMP. The conditions were successfully applied to the hydrodefluorination of biologically relative molecules, such as a prostate cancer drug bicalutamide, a cannabinoid receptor agonist BAY 59-3074, a mild heart failure and hypertension drug bendroflumethiazide, and a hormonal therapy drug enzalutamide. However, dual hydrodefluorination of the ArCF_3_ to give ArCH_2_F is always observed as a side reaction because mono-hydrodefluorination products ArCF_2_H have weaker C-F bonds while slightly negative redox potentials.

**Scheme 18:**
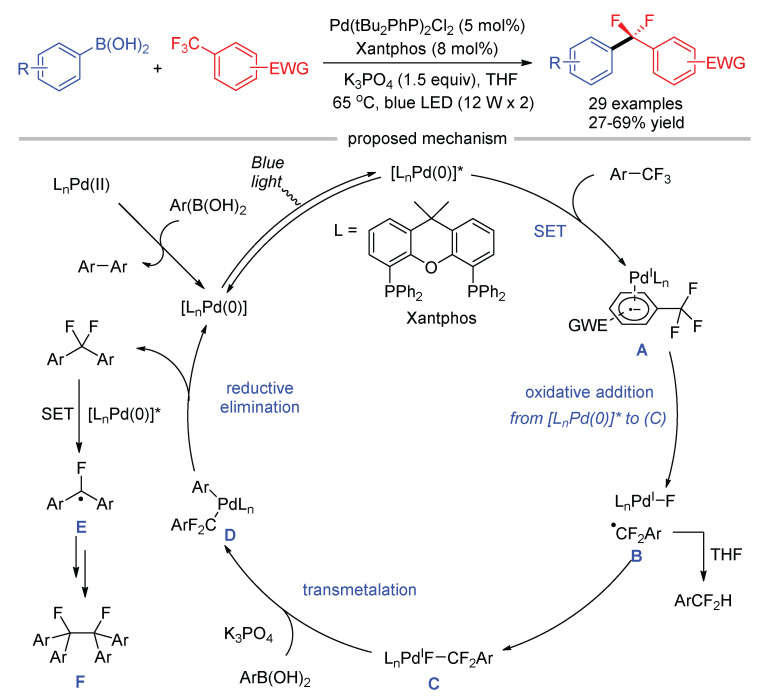
Visible-light-induced palladium-catalyzed defluoroarylation of trifluoromethylarenes with arylboronic acids.

A visible light-induced palladium-catalyzed selective defluoroarylation of trifluoromethylarenes with arylboronic acids was recently disclosed by the Zhang group via the Pd(0)-Pd(I)-Pd(II) catalytic cycle (Scheme 18) [59]. The reaction used Pd(*^t^*Bu_2_PhP)_2_Cl_2_ as the catalyst and Xantphos as the ligand. However, a new palladium complex Pd(Xantphos)_2_ was demonstrated as the active species for promoting the reaction, whose oxidation potential was calculated to be $E_{1/2}^{\left(\left[\mathrm{PdI}\right]/\left[\mathrm{Pd}0\right]*\right)}$ = −2.76 V vs. Fc^+^/Fc in THF. Therefore, the SET reduction of trifluoromethylarenes by an excited-state [Pd(0)L_n_]* generates α,α-difluororobenzylic radical **B** and [F-Pd(I)L_n_] species through C–F bond cleavage. The recombination of **B** and [F-Pd(I)L_n_] forms [ArCF_2_-Pd(II)FL_n_] complex **C**. Transmetalation of **C** with arylboronic acid produces [ArCF_2_-Pd(II)L_n_-Ar] **D**, which undergoes reductive elimination to deliver defluoroarylation product and regenerates Pd(0) catalyst. The proposed mechanism gave a reasonable explanation for the formation of side products, such as the ArCF_2_H generated by the hydrogen abstraction of α,α-difluororobenzylic radical **B** from solvent THF and over-defluorination for the formation of dimerization products **F**.

**Scheme 19:**
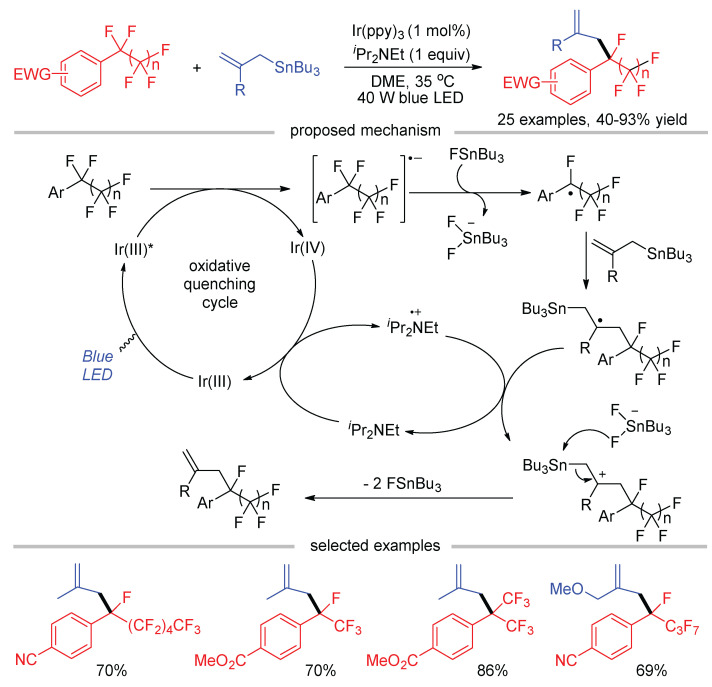
Photoredox-catalyzed C–F bond allylation of perfluoroalkylarenes.

Nishimoto and Yasuda reported the site-selective C-F bond activation of perfluoroalkylarenes at the benzylic position through perfluoroalkyl radicals generated by photoredox catalysis (Scheme 19) [60]. Prolonging the carbon chain of the perfluoroalkyl group not only decreases the stability of the radical and but also increases the steric hindrance to inhibit subsequent bond formation. As a result, the reaction tends to return to its starting point via the reverse F^−^ addition and back electron transfer (BET). To overcome these problems, a highly reactive allylic stannane was used as the coupling partner to prevent the retro-reaction, which generates Bu_3_SnF in situ to trap F^−^ by forming thermodynamically stable Bu_3_SnF_2_^−^. The Stern–Volmer luminescence quenching experiments indicated that *^i^*Pr_2_Net and allylic stannane exhibited less efficient quenching effects for the excited state of Ir(ppy)_3_ in comparison with perfluoroalkylarenes. Therefore, a mechanism involving the oxidative quenching of excited Ir(ppy)_3_ by perfluoroalkylarenes was proposed. Various perfluoroalkyl groups, such as linear Ar–*^n^*C_4_F_9_ and Ar–*^n^*C_6_F_13_, as well as heptafluoroisopropylarenes (Ar–CF(CF_3_)_2_) underwent site-selective defluoroallylation regardless of the large steric hindrance.

**Scheme 20:**
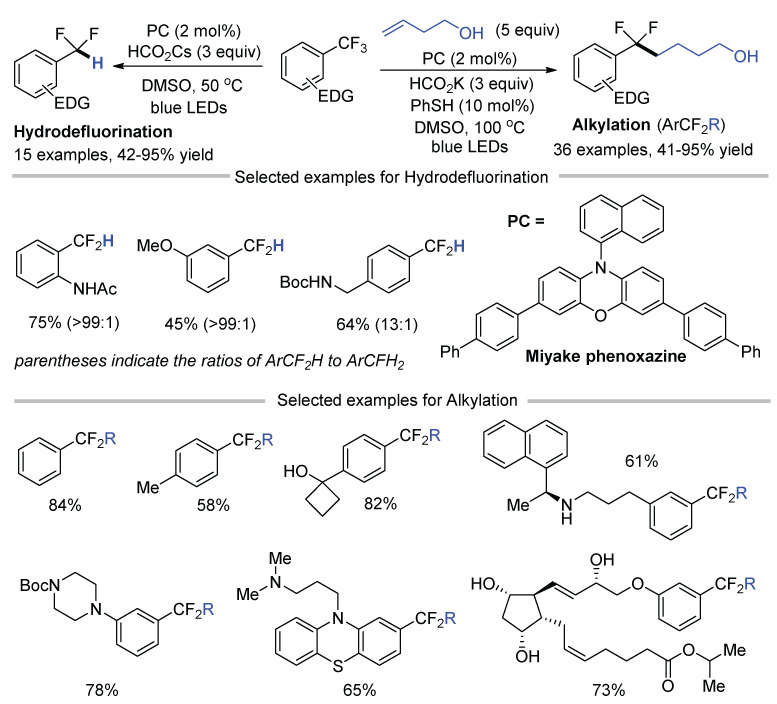
Hydrodefluorination and defluoroalkylation of unactivated ArCF_3_.

The presence of an electron-withdrawing group on the aromatic ring of trifluoromethylarenes can increase the reduction potential of the substrates, thus rendering them more reactive to accept an electron for C–F bond cleavage via an SET pathway. Trifluoromethylarenes bearing an additional CF_3_ group or auxiliary CN, ester, and –SO_2_NH_2_ groups are typical substrates used in the above-mentioned reactions. Jui reported the C-F functionalization of trifluoromethylarenes substituted by electron-rich groups at an elevated temperature using a phenoxazine-based photocatalyst (Scheme 20) [61]. Difluorobenzylic radicals, generated by the C-F cleavage of the simplest benzotrifluoride or various alkyl-, oxygen-, and nitrogen-substituted trifluoromethylarenes, can be utilized in alkylation process with a series of olefins as the radical acceptors. In the absence of olefins, direct hydrodefluorination occurred efficiently under slightly modified conditions.

## 5. Trifluoromethyl Alkenes

**Scheme 21:**
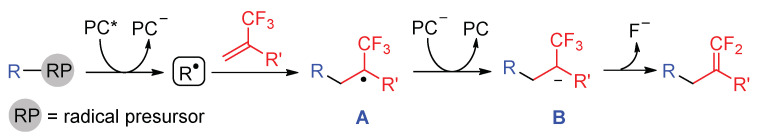
General mechanism for the photocatalytic C-F bond of CF_3_-alkenes.

The C-F bond cleavage of trifluoromethyl alkenes usually proceeds through the radical addition and fluoride elimination pathway. Alkenes are good radical acceptors while the attachment of a CF_3_ group further lowers the LOMO of the C-C double bond, making radical addition to alkenes easier. To access the α-CF_3_ carbon ion **B** necessary for fluoride elimination, the excited photocatalyst (PC*) must be reductively quenched by a radical precursor to give PC^−^ and radical R^•^. The addition of R^•^ to α-CF_3_ alkenes provide α-CF_3_ alkyl radical **A**, which is SET reduced by PC^−^, affording anionic intermediate **B** concomitant with the regeneration of ground-state photocatalyst (PC). Finally, β-fluoride elimination from **B** leads to *gem*-difluoroalkene products (Scheme 21).

**Scheme 22:**
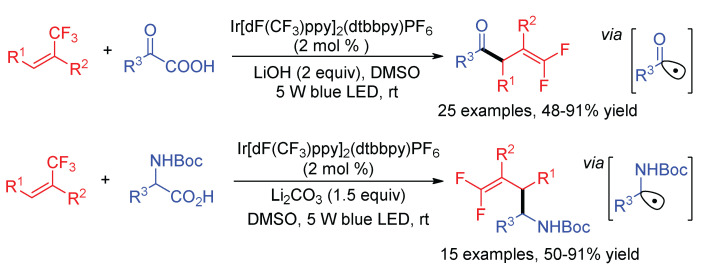
Synthesis of functionalized *gem*-difluoroalkenes.

This photocatalytic radical-polar crossover strategy for the synthesis of functionalized *gem*-difluoroalkenes was first reported by our group in 2016 [62]. Various γ,γ-difluoroallylic ketones were readily obtained in good yields by a photocatalytic decarboxylative/defluorinative reaction of α-ketoacids and α-trifluoromethyl alkenes (Scheme 22). In addition, using α-amino acids as the precursor of α-amino alkyl radicals, the slightly modified reaction conditions can be used to synthesize 1,1-difluorohomoallyl amines, which were previously prepared by five steps from CF_3_CH_2_OTs [63].

**Scheme 23:**
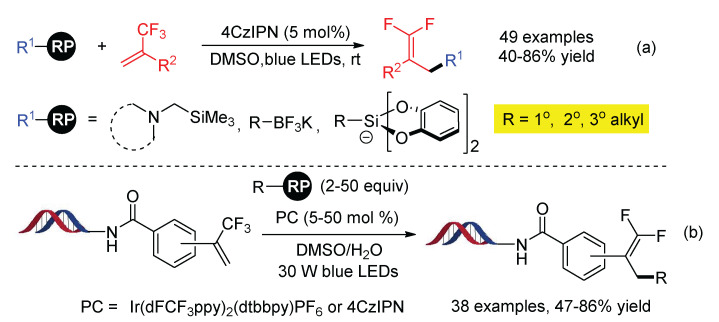
(**a**) Defluoroalkylation of trifluoromethyl alkenes and (**b**) applications in DNA-encoded library synthesis.

Subsequently, α-trimethylsilylamines, potassium organotrifluoroborates, and alkylbis(catecholato)silicates were applied as the precursors of alkyl radicals in the C-F cleavage of trifluoromethyl alkenes by Molander (Scheme 23a) [64]. Due to the strong coordination of Si or B with F^−^, base is not needed, allowing the reaction to proceed at near-neutral pH. In addition, an inexpensive organic photocatalyst 4CzIPN was effective for the reaction. Taking advantage of these benefits, the reaction was further applied to enrich the DNA-encoded library via the photocatalytic defluorinative coupling of DNA-tagged α-CF_3_ styrenes and alkyl radical precursors in the open air and aqueous media (Scheme 23b) [65].

**Scheme 24:**
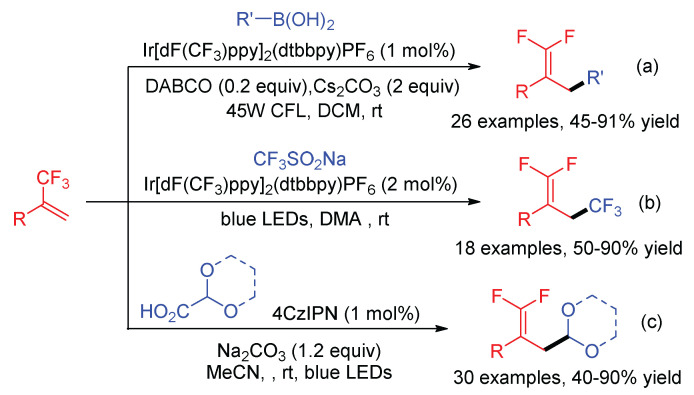
(**a**) Alkylboronic acids, (**b**) CF_3_SO_2_Na and (**c**) glyoxylic acid acetals as the radical precursors for the photocatalytic defluoronative coupling with CF_3_-alkenes.

The robustness of this visible-light-mediated radical-polar crossover addition–elimination protocol for the C-F bond cleavage of α-trifluoromethyl alkenes was further demonstrated by the use of alkylboronic acids (Scheme 24a) [66], CF_3_SO_2_Na (Scheme 24b) [67], and glyoxylic acid acetals (Scheme 24c) [68] as the radical precursors. The common feature of the radical sources is their low redox potential to be SET oxidized by excited photocatalyst, producing a one-electron-reduced form of photocatalyst (PC^−^) to switch the subsequent α-CF_3_ carbon radicals to carbon ions.

**Scheme 25:**
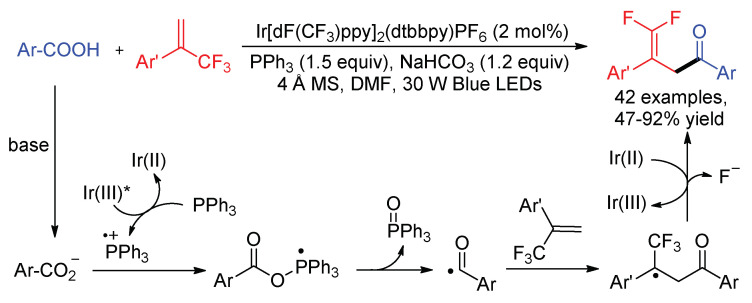
Deoxygenation/defluorination protocol for the synthesis of γ,γ-difluoroallylic ketones.

As mentioned in Scheme 22, we reported the synthesis of γ,γ-difluoroallylic ketones by the reaction of α-ketoacids and α-trifluoromethyl alkenes, in which acyl radicals were generated from α-ketoacids by SET oxidation and the extrusion of CO_2_. These compounds can also be prepared using readily available aryl carboxylic acids as the acyl radical precursors through the direct deoxygenation of acids in the presence of PPh_3_ (Scheme 25) [69]. In this reaction, PPh_3_ $E_{\mathrm{red}}^{\left(1/2\right)}$ = +0.98 V vs. SCE) acted as the electron donor to reductively quench excited Ir(III)*, providing a PPh_3_ radical cation and an Ir(II) species. The reaction of radical cation with carboxylates generated phosphoryl radical, which underwent β-C–O bond cleavage to form triphenylphosphine oxide and acyl radicals.

**Scheme 26:**
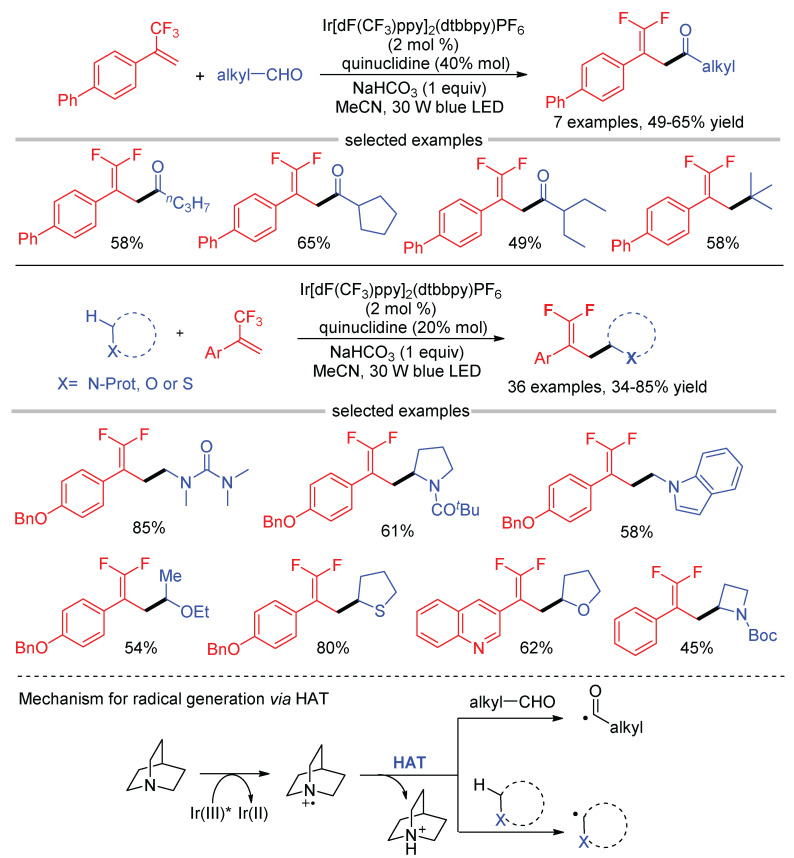
α-C–H difluoroallylation by photoredox/hydrogen atom transfer.

The HAT of aldehydes is also a useful method to generate acyl radicals. Wang developed a photocatalytic C–H difluoroallylation of alkyl aldehydes using quinuclidine as the HAT catalyst (Scheme 26) [70]. The reaction led to γ,γ-difluoroallylic alkyl ketones in moderate yields. However, in the case of tertiary aldehydes, only the decarbonylated products were obtained, driven by the decarbonylation of the acyl radicals to form more stable tertiary alkyl radicals. In addition, aryl aldehydes and alkyl aldehydes bearing a benzene ring were not suitable substrates for this reaction. Except for the C(sp^2^)-H bond in the aldehydes, the activation of C(sp^3^)–H adjacent to a heteroatom, such as N, O, and S, is more effective, offering a convenient method to convert amides, ethers, and thioethers into the corresponding γ,γ-difluoroallylic compounds in good yields via the combination of HAT and photoredox catalysis.

**Scheme 27:**
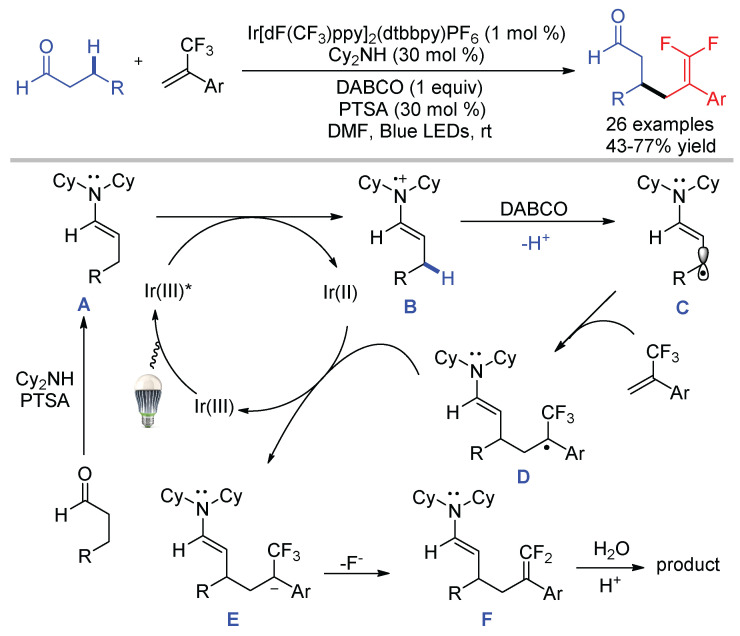
β-C–H *gem*-difluoroallylation of aldehydes.

Our group reported the visible-light-mediated β-C–H difluoroallylation of aldehydes through C–F bond cleavage of α-trifluoromethyl alkenes [71]. In contrast to the generation of acyl radicals via HAT, the synergistic combination of photoredox catalysis and organocatalysis produced β-enaminyl radicals by the activation of β-C–H of aldehydes. As shown in Scheme 27, the condensation of aldehyde and Cy2NH in the presence of *p*-toluenesulfonic acid (PTSA) gave enamine **A**, which was single-electron oxidized by Ir(III)* to form radical cation **B** and Ir(II). The acidic β-C–H of radical cation **B** was deprotonated by DABCO, producing β-enaminyl radical **C** for the subsequent defluorinative coupling with α-trifluoromethyl alkenes. The hydrolysis of difluoroallylated enamine **F** regenerated organocatalyst Cy2NH and uncovered the carbonyl group.

**Scheme 28:**
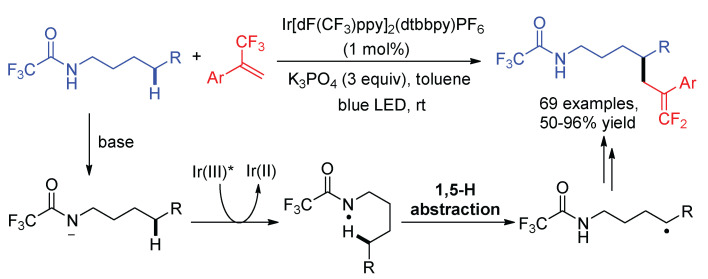
δ-C–H *gem*-difluoroallylation of amides via intramolecular 1,5-HAT.

Regioselective difluoroallylation of remote inactivated C(sp^3^)–H bond in amides was developed via an intramolecular 1,5-HAT process (Scheme 28) [72]. Martin disclosed the δ-C–H difluoroallylation of secondary trifluoroacetamides with α-trifluoromethyl styrenes using Ir(dFCF_3_ppy)_2_(dtbbpy)PF_6_ as the photocatalyst. In the presence of base, the deprotonation of trifluoroacetamides followed by single-electron oxidation generated an amidyl radical for the 1,5-HAT. The electron-withdrawing CF_3_ group in the amides is important for this reaction, which not only acidified the N–H bond but also stabilized the *N*-centered radicals. Pan independently reported the same reaction using 4CzIPN as the photocatalyst [73].

**Scheme 29:**
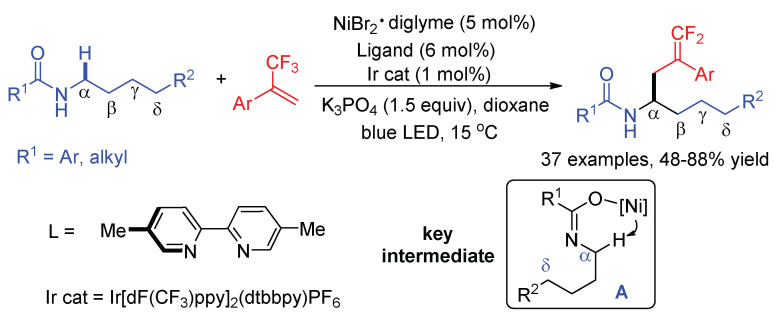
α-C–H *gem*-difluoroallylation of amides in the combination of nickel and photoredox catalysis.

Interestingly, Martin found that the site selectivity of difluoroallylation switched from the δ-C–H bond of amides to the α-position when catalytic amounts of NiBr_2_·diglyme and 5,5′-dimethyl 2,2′-bipyridine were added (Scheme 29) [72]. The formation of an intermediate **A** by binding Ni(II) precatalyst to the oxygen site of amide was the reason for the activation of the α-C–H bond. Aryl- or alkyl-substituted secondary amides were more effective substrates for this transformation.

**Scheme 30:**
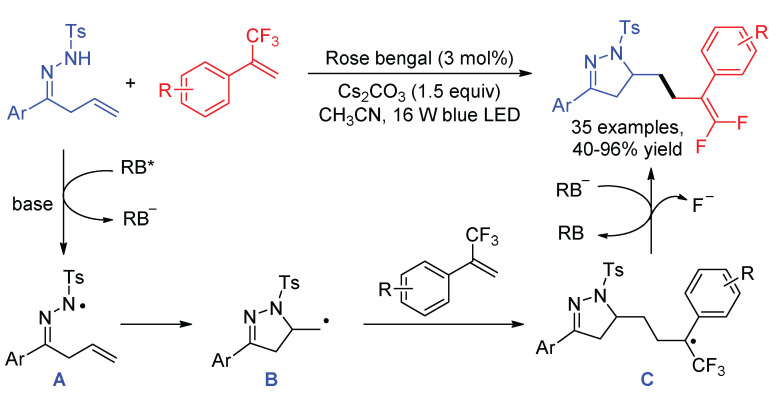
Synthesis of dihydropyrazole-fused *gem*-difluoroalkenes.

The *5-exo-trig* radical cyclization is a useful method to convert *N*-centered radicals to carbon radicals [74]. Zhou and co-workers described the synthesis of dihydropyrazole-fused *gem*-difluoroalkenes via the visible-light-promoted reactions of β,γ-unsaturated hydrazones and α-trifluoromethyl alkenes using organic dye Rose Bengal (RB) as the photocatalyst (Scheme 30) [75]. With the assistance of base single-electron oxidation of *N*-tosyl β,γ-unsaturated hydrazones by excited RB* forms *N*-center hydrazonyl radicals **A**, which undergo addition to the terminal C-C double bond intramolecularly. The resultant C-centered radicals **B** attack the α-trifluoromethyl alkenes, followed by single-electron reduction and β-fluoride elimination to afford the *gem*-difluoroalkene products.

**Scheme 31:**
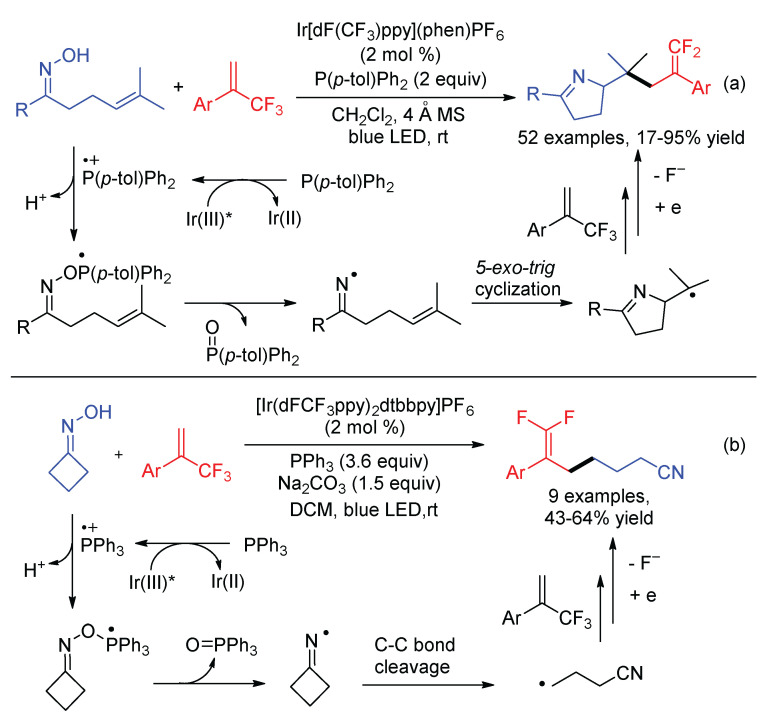
Generation of iminyl radicals via phosphoranyl radical-mediated N–O cleavage of oximes: (**a**) intramolecular cyclization; (**b**) C–C bond cleavage.

Iminyl radicals are another type of *N*-centered radicals that are widely used in the organic synthesis [76]. Using triarylphosphine as the deoxygenative reagents, iminyl radicals can be generated from oximes directly via the photocatalytic phosphoranyl radical-mediated N-O bond cleavage. When this method was applied to γ,δ-unsaturated oximes, iminyl radicals trigger further intramolecular radical cyclization to form alkyl radicals bearing the 2H-pryrole motif, which then undergo defluoronative reaction with trifluoromethyl alkenes (Scheme 31a) [77]. While using strained cyclobutanone oxime as the substrates, the iminyl radical generated by deoxygenation underwent C-C bond cleavage to give γ-cyano alkyl radical, which allowed the synthesis of *gem*-difluoroalkenes bearing a cyano group (Scheme 31b) [78].

**Scheme 32:**
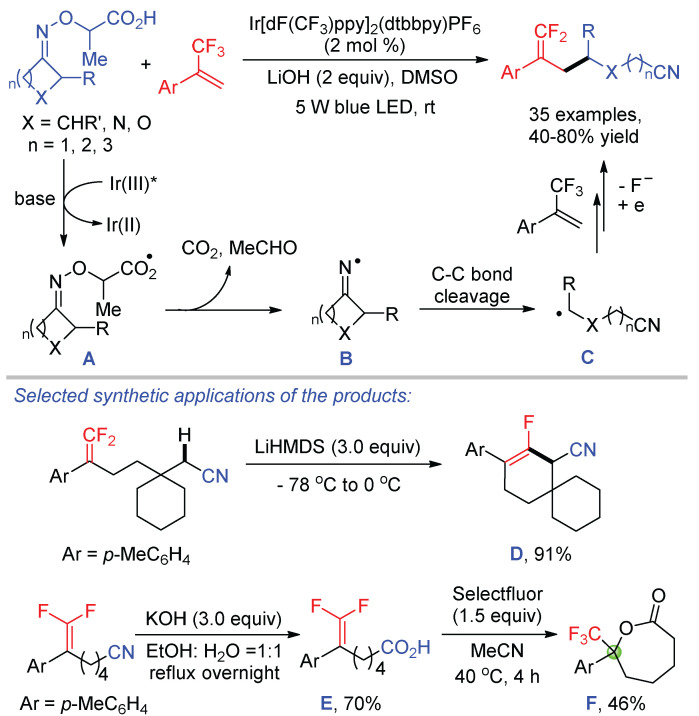
Redox neutral γ,γ-difluoroallylation of cycloketone oxime ethers.

As a continuation of our interesting in decarboxylative/defluorinative reaction, our group reported a visible-light-promoted redox neutral γ,γ-difluoroallylation of cycloketone oxime ethers with trifluoromethyl alkenes, which also afforded various *gem*-difluoroalkenes bearing a cyano group (Scheme 32) [79]. By introducing a carboxylic group into the oxime ethers, single-electron oxidation of carboxylates followed by extrusion of one molecule of CO_2_ and MeCHO produced iminyl radicals **B** for the subsequent C-C bond cleavage. *gem*-Difluoroalkenes and nitriles are two of the most ubiquitous chemical feedstocks in organic synthesis. It is of significance to introduce these two groups into one molecule, as demonstrated by the synthesis of cyclic monofluoroalkene **D** via a second C–F bond cleavage and ε-caprolactone **E** via an intramolecular fluoro-functionalization.

**Scheme 33:**
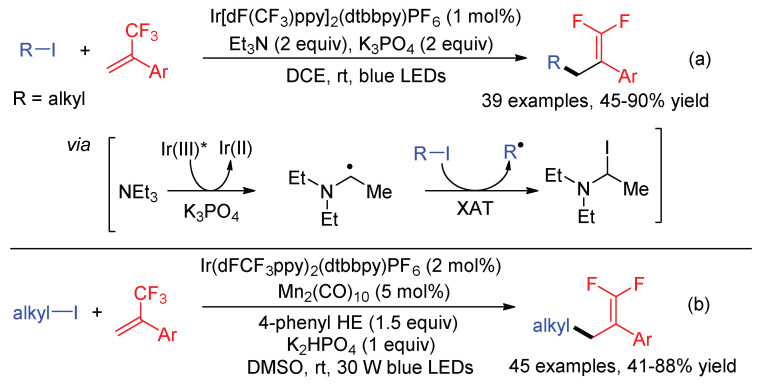
Photocatalytic reductive coupling of alkyl iodides with CF_3_-alkenes: (**a**) XAT mediated by an α–aminoalkyl radical; (**b**) XAT mediated by Mn_2_(CO)_10_.

Single-electron reduction of alkyl halides by an excited-state photocatalyst has been developed as a powerful tool to generate alkyl radicals. However, only the activated C-X bond, which is usually adjacent to one or two strong electron-withdrawing groups, can be reduced, while inactivated alkyl iodides are difficult to participate in redox chemistry due to their high reduction potentials (*E*_red_ < 2 V vs. SCE) [80]. In addition, applying alkyl halides in the defluorinative coupling with trifluoromethyl alkenes needs the addition of a reducing reagent because of the release of both X^−^ and F^−^ during the reaction. Wang developed visible-light promoted C–I difluoroallylation reactions of trifluoromethyl styrenes and alkyl iodides with an α-aminoalkyl radical as the mediator (Scheme 33a) [81]. NEt_3_ acts as the electron donor to single-electron reduce excited Ir photocatalyst, affording Ir(II) and α-aminoalkyl radical, while the resultant α-aminoalkyl radical abstracts iodine atoms from alkyl iodides to generate open-shell alkyl radicals via halogen-atom transfer (XAT). Although the direct addition of α-aminoalkyl radicals to trifluoromethyl styrenes has been reported [62,64], this side reaction was not observed. Presumably due to the low BDE of the C-I bond, α-aminoalkyl radicals might undergo fast XAT with alkyl iodides prior to trapping by α-trifluoromethyl styrenes. Subsequently, Wang also reported the same reaction using Mn_2_(CO)_10_ as the XAT catalyst and 4-phenyl Hantzsch ester (HE) as the reducing reagent (Scheme 33b) [82].

**Scheme 34:**
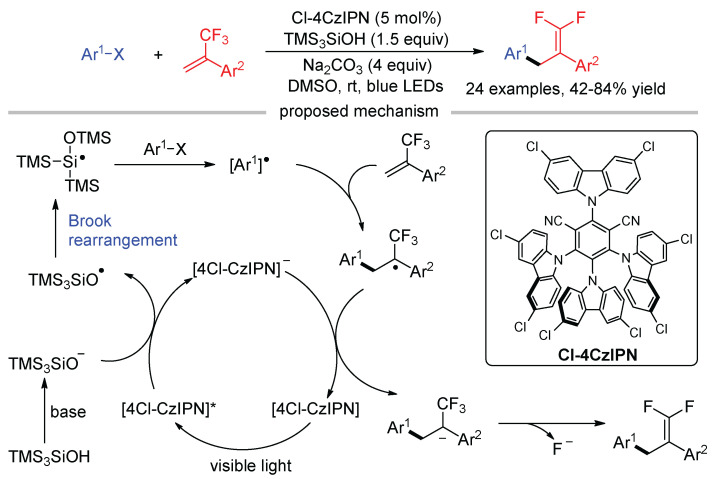
Defluorinative arylation of trifluoromethyl alkenes.

Molander reported the first defluorinative arylation of trifluoromethyl alkenes, in which aryl radicals were generated from aryl halides with tris(trimethylsilyl)silanol (TMS_3_SiOH) as the XAT reagent (Scheme 34) [83]. Base-mediated deprotonation of TMS_3_SiOH gives TMS_3_SiO^−^, which was oxidized by the photocatalyst followed by a radical Brook rearrangement to form silyl radical. Then, the silyl radical abstracts a halogen atom from aryl halides, generating aryl radicals. The reaction used Cl-4CzIPN as the photocatalyst, which has higher oxidative ability (*E_p_* = +1.71 V vs. SCE) than 4CzIPN (*E_p_* = +1.35 V vs. SCE) due to the introduction of two electron-withdrawing chloro groups to the 3,6-positions of carbazole motifs.

**Scheme 35:**
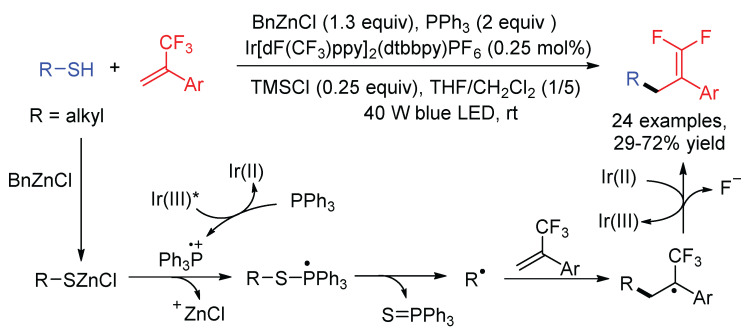
Desulfurative generation of alkyl radicals from thiols.

As described above, phosphinyl radical cations have been employed for the deoxygenative activation of sodium sulfinates (Scheme 16), carboxylic acids (Scheme 25), and oximes (Scheme 31). Recently, Dilman disclosed a novel approach to generate alkyl radicals via phosphine-mediated C-S bond cleavage of thiols, which react with α-CF_3_ styrenes to give *gem*-difluorostyrenes (Scheme 35) [84]. Thiol is known as a good hydrogen donor in radical reactions. To avoid the facile hydrogen atom transfer (HAT) between thiols and latter generated radicals, thiols were first converted to the corresponding zinc thiolates by their fast reactions with benzyl zinc chloride. Then, the interaction of the zinc thiolates with PPh_3_ in the presence of oxidative photocatalyst generates neutral sulfur-phosphorus radicals, which afford alkyl radicals by the elimination of S=PPh_3_. Radical addition, SET reduction by Ir(II), and fluoride elimination provided *gem*-difluoroalkene products. The use of 0.25 equivalent of chlorotrimethylsilane (TMSCl) was essential for reproducibility, although its precise role is not clear. These phosphine-mediated deoxygenative or desulfurative reactions with trifluoromethyl alkenes significantly expand the scope of radical precursors for photocatalytic reductive cross-coupling.

**Scheme 36:**
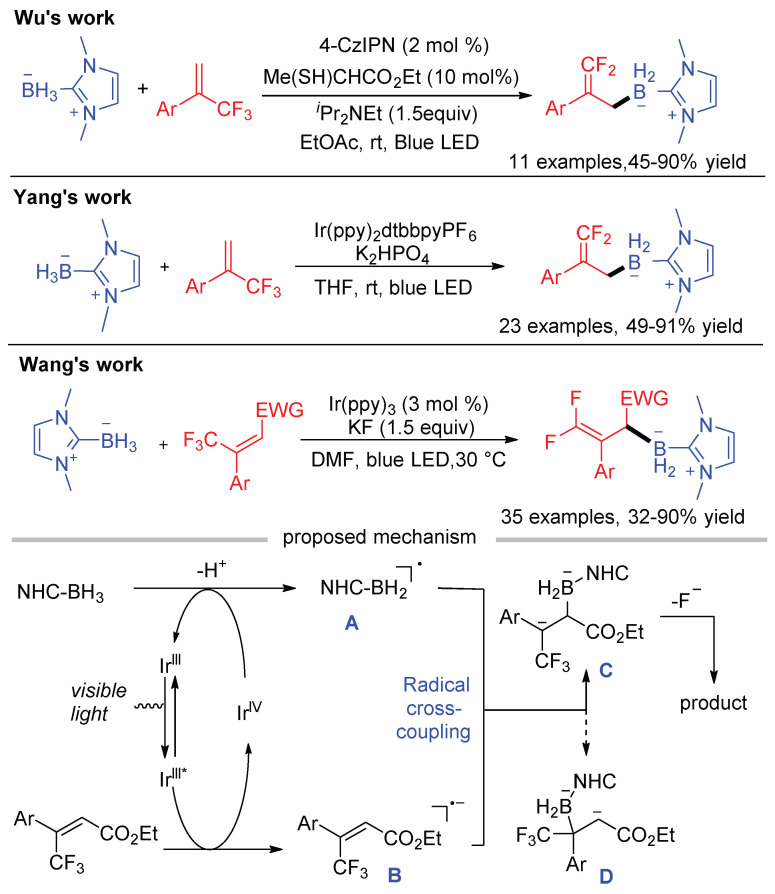
Radical defluoroborylations of trifluoromethyl alkenes.

Boron-centered radicals are important reactive intermediates in organic synthesis. Wu [42], Yang [85], and Wang [86] independently reported the radical defluorinative borylations of trifluoromethyl alkenes by visible light photoredox catalysis (Scheme 36). Although all these reactions afforded the *gem*-difluoroallylborane products, their mechanisms are a little different. Wu proposed a HAT process for the generation of NHC-boryl radical (see Scheme 7), while the same radial is generated via the direct oxidation of NHC-BH_3_ by excited Ir(III) species followed by deprotonation in Yang’s work (see Scheme 8). The subsequent radical addition, SET reduction, and β-fluoride elimination were ensuring transformations in both works. In contrast, the cross-coupling of NHC-boryl radical **A** and radical anion **B** was suggested as the key step to form the α-CF_3_ carbon ion **C** in Wang’s protocol. Due to the presence of an additional electron-withdrawing group (EWG) in the β-position of α-CF_3_ styrenes, these substrates are easier to be SET reduced by Ir(III)* ($E_{1/2}^{\mathrm{Ir}\left(\mathrm{III}\right)/\mathrm{Ir}(\mathrm{IV})}$ = −1.73 V vs. SCE), providing radical anions **B** and Ir(IV). Then, NHC-BH_3_ (*E*_1/2_ = + 0.76 V vs. SCE) was oxidized by Ir(IV) ($E_{1/2}^{\mathrm{Ir}\left(\mathrm{III}\right)/\mathrm{Ir}(\mathrm{IV})}$ = + 0.77 V vs. SCE) to give NHC-boryl radical **A** after the deprotonation. The presence of EWG on the C-C double of trifluoromethyl alkenes and the use of *fac*-Ir(ppy)_3_, a strongly reducing photocatalyst in its excited state, are crucial for the formation of radical anion **B**. The cross-coupling of NHC-boryl radical **A** and radical anion **B** occurred at sterically less hindered α-carbon, which has lower activation Gibbs energy than the reaction at the β-carbon according to the DFT calculations. Such radical–radical combination provides the α-CF_3_ carbon ion **C**, which undergoes β-fluoride elimination to form the borylation products.

**Scheme 37:**
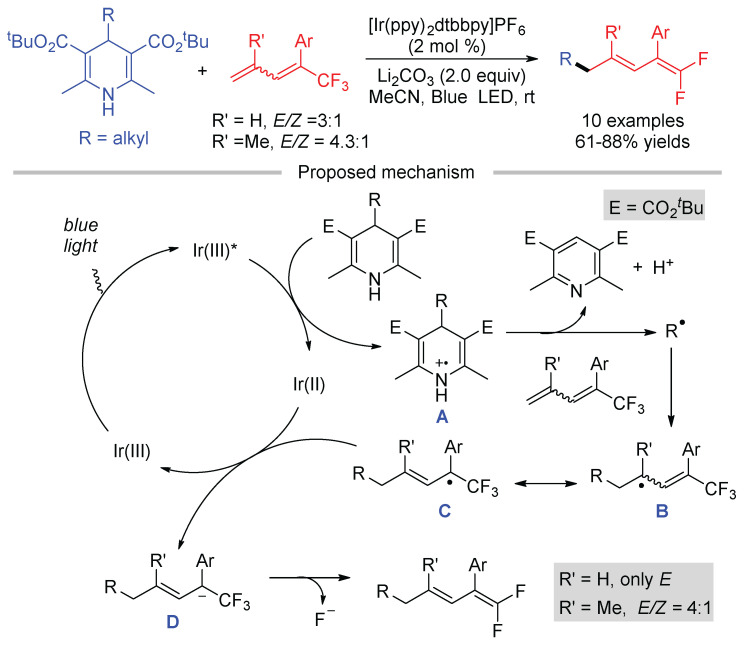
Defluorinative alkylation of 1-trifluoromethyl 1,3-butadienes.

Although diverse radical precursors have been developed for the defluorinative coupling with trifluoromethyl alkenes, little effort has been devoted to the C-F bond cleavage of the CF_3_ group attached to other conjugated π-systems. Our group reported the first photoredox defluorinative alkylation of 1-trifluoromethyl 1,3-butadienes with 1,4-dihydropyridines as the alkylation reagents [87]. The reaction took advantage of the resonant structures of allyl radicals. As shown in Scheme 37, the reduction of 4-alkyl 1,4-dihydropyridines by excited Ir(III) gives radical cation **A**. Driven by the formation of an aromatic pyridine, radical cation **A** undergoes the C-C bond to deliver alkyl radical. The addition of allyl radical to 1-trifluoromethyl 1,3-butadienes forms allyl radical **B**, which would tautomerize to a more stable allyl radical **C** due to the presence of both the aryl and CF_3_ group. Then, the reduction of α-CF_3_ allyl radical **D** by Ir(II) and β-fluoride elimination afford the desired 1,1-difluorodienes. The diastereoselectivity of the products was not significantly affected by the *E*/*Z* ratio of the 1,3-butadienes. When R′ = H, only *E*-isomers of 1,1-difluorodienes were obtained after the defluorinative alkylation.

**Scheme 38:**
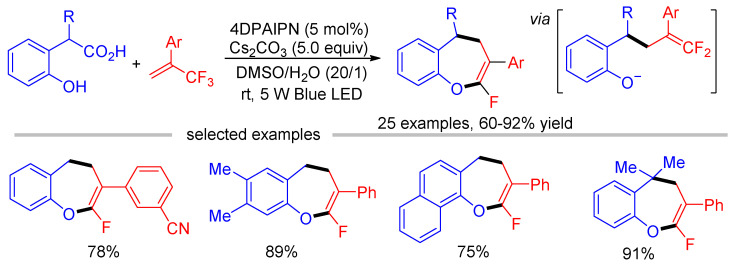
Synthesis of fluorinated dihydrobenzoxepines via dual C-F bond cleavage.

The cleavage of one C-F bond of trifluoromethyl alkenes via the radical-polar cross over strategy allows the facile synthesis of various *gem*-difluoroalkens. As described in Section 3, *gem*-difluoroalkens can also undergo defluorinative coupling with radical precursors under visible light conditions. We have previously reported the [3 + 3] annulation of trifluoromethyl alkenes with tertiary amines [88], or dihydroisoquinoline acetic acids [89] via dual C-F bond cleavage in a CF_3_ group using a single photocatalyst. However, this protocol was limited to the synthesis of *N*-containing heterocyclic compounds because α-aminoalkyl radicals are necessary intermediates for both steps of C–F bond cleavage. In the combination of the photoredox catalysis and base-mediated S_N_V reaction, our group developed a photocatalytic decarboxylative/defluorinative [4 + 3] annulation of *o*-hydroxyphenylacetic acids and trifluoromethyl alkenes (Scheme 38) [90]. The first photocatalytic C-F bond cleavage of trifluoromethyl alkenes led to *gem*-difluoroalkenes, which were readily converted into fluorinated dihydrobenzoxepines by cleaving a second C-F bond in the presence of base.

## 6. Conclusions

Selective C-F bond cleavage of readily available per- or poly-fluorinated compounds has emerged as a powerful tool for effective synthesis of fluoroorganic compounds. In this review, we summarized the advances in C-F bond cleavage of polyfluorinated arenes, *gem*-difluoroalkenes, trifluoromethyl arenes, and trifluoromethyl alkenes enabled by visible light photoredox catalysis from 2018 to the present. The level of interest in this area is growing rapidly, because the reactions in this review trebled those in our previous book chapter with the same focus. If electrochemical methods and transition metal-catalyzed reductive coupling reactions were counted, the progress on radical-involved C-F bond cleavage is even greater. Visible-light-promoted C-F bond cleavage, just like other easily handling reactions by photoredox catalysis, obtained a significant fillip because visible light is mild, green, and infinitely available. Photocatalysts in their excited states can be either single-electron oxidized or single-electron reduced. Due to their unique redox property and moderate redox potentials, visible light photocatalysts are expected to find more applications in selective cleavage of the C-F bond.

Since this field is currently shaping up, there is a lot more that needs to be explored by chemists in the future especially in terms of the broad scope of poly-fluorinated substrates. For example, an additional electron-withdrawing group on the benzene ring is usually needed for the C-F bond cleavage of poly-fluorinated arenes and trifluoromethyl arenes; at least one aryl group is necessary for the efficient C-F bond cleavage of *gem*-difluoroalkenes, α-CF_3_ styrenes are the most common substrates for the defluorinative coupling with various radical precursors while examples of α-CF_3_ alkenes bearing one or two substituents on the β-positions are rare. Except for the C-F bond attached to the C-C double and aromatic rings, readily available poly-fluorinated reagents bearing other π-systems, such as trifluoroacetyl derivates and trifluoromethyl acetylenes, have not achieved by visible light photocatalysis [91]. The C-F activation of fluorinated compounds without any π-system is still the most challenging task, even using organometallic reagents or main group Lewis acids under harsh conditions. The combination of visible light photoredox catalysis with transition metal catalysis or electrocatalysis might be a solution for this issue. Overall, with the development of a selective C-F bond of more and more poly-fluorinated compounds, we can anticipate that this strategy will continue to make important contributions in organic synthesis and find more applications in medicinal chemistry and material science.

## Data Availability

All data are available in the manuscript.
